# Gamified Environments and Serious Games for Students With Autistic Spectrum Disorder: Review of Research

**DOI:** 10.1007/s40489-023-00381-7

**Published:** 2023-05-11

**Authors:** Nerea López-Bouzas, M. Esther del Moral-Pérez

**Affiliations:** University of Oviedo, Oviedo, Spain

**Keywords:** Gamified Environments, Serious Games, Autistic Spectrum Disorder, Revision of research

## Abstract

The aim of this study is to review the available research (*N* = 70) derived from the use of Gamified Environments and Serious Games with people with Autistic Spectrum Disorder (ASD), identifying: authorship, nationality, publication period, topic, and design of the investigation. After that, the advantages and limitations observed are identified. Results indicate that most of them are focused on the design and testing of prototypes, (mostly) linked to the increase of social and emotional skills. The revision highlights that the game’s mechanics and dynamics (feedback, rewards, missions, etc.) involve students from motivation. There is unanimous agreement to emphasize the positive impact of these resources to increment self-control, self-conscience, autonomy, and empathy.

## Introduction

There are increasingly more educational practices that include technological resources and games (Hashim, 2018). According to Torres et al. (2018), games with computer interfaces and training applications games with computer interfaces and training applications differ from the conventional videogame because they have an educational purpose. These educational games can be grouped following three typologies: *simulation games*, aimed at creating situations similar to real ones for training certain skills; *Serious Games (SG)*, designed from a narrative which invites the player to overcome obstacles by testing his/her knowledge and skills (Dörner et al., 2016); and, digital *gamified environments*, scenarios which turn the teaching-learning process into a game by adopting the mechanics, dynamics, and aesthetics of the video game (Torres and Romero, 2019). The difference between SG and digital gamified environments is that the first ones constitute complete and closed digital games, while digital gamified environments are open scenarios that combine playful elements and activities which appropriate the qualities of video games (Deterding et al., 2011).

The integration of the different types of digital gamified environments and SG in the educational context is increasingly noticeable, also in the field of Special Education. Specifically, people with Autism Spectrum Disorder (ASD) tend to prefer digital resources over traditional ones in order to interact with educational purposes, since their structure and support is adjusted — to a greater extent — to their cognitive processes (Grossard et al., 2017). On the other hand, both the use of gamified environments and SG allow to increase their motivation in therapeutic sessions, managing to improve long-term behaviors (Malinverni et al., 2017; Van Dooren et al., 2019). Specifically, Gaudi et al. (2020) consider that SG are becoming an effective alternative to traditional therapy, since they allow adaptation to specific needs and individual casuistry by covering a wide range of scenarios and characters.

These resources make the adaptation of the content to the individual characteristics easier to the students, regardless of their growth rate, and, furthermore, allow for everyday simulations of the real world (Grossard et al., 2019a, b; Mubin et al., 2020). In addition, digital environments supported by games with visual stimulate the assimilation and significance of the experience, increasing learning and reducing the cognitive load (Lozano et al., 2012; Wang et al., 2022). Thus, the gamification of activities in digital environments and the SG dispense rewards that facilitate individual progress according to the maturational rhythms of people with ASD (Kientz et al., 2013).

In addition, the embodiment of narratives and characters from the real world in digital environments allows to minimize problems related to the acquisition of new learning, being a source of stimulation of interpersonal relationships among student players (Griffin et al., 2021; Malinverni et al., 2017; Politis, 2017). The proliferation of interventions with people with ASD supported by the use of SG and gamified environments is achieving successful results (Arzone et al., 2020; Griffin et al., 2021; Mota et al., 2020; Scherf et al., 2018; Terlouw et al., 2021); thus, the aim of this study is to carry out a systematic review of the publications derived from this type of experiences carried out between 2017 and 2022, identifying the advantages and limitations to infer their suitability and facilitate their dissemination so that these experiences can be carried out in other contexts, minimizing those aspects which could have obstructed their suitable development.

## Methods

This systematic review has been developed taking into account the principles of the PRISMA declaration (Moher et al., 2015) and the standardized methodological guidelines proposed for the preparation of quality systematic reviews (Alexander, 2020). Between January and February 2023, a Google Scholar search was carried out, which was limited to selecting articles that met the following criteria: (1) research on the use of gamified environments and/or Serious Games in students with previously diagnosed ASD; (2) studies which are in databases such as SAGE, SCOPUS, Springer LINK, Web of Science, Wiley Online Library, Oxford Journals, Cambridge Journals, Google Scholar, and Science Direct; and (3) international publications and in English that have been published between since there are already other revisions prior to that interval. The aim is to update the literature review after observing the proliferation of studies focused on the use of digital environments and Serious Games in students with ASD in the last 5 years, period in which this funded research is framed. The following keywords were used for the search: “ASD” or “Autism” + “Gamification”/“ASD” or “Autism” + “Serious Games.”

**Fig. 1:**
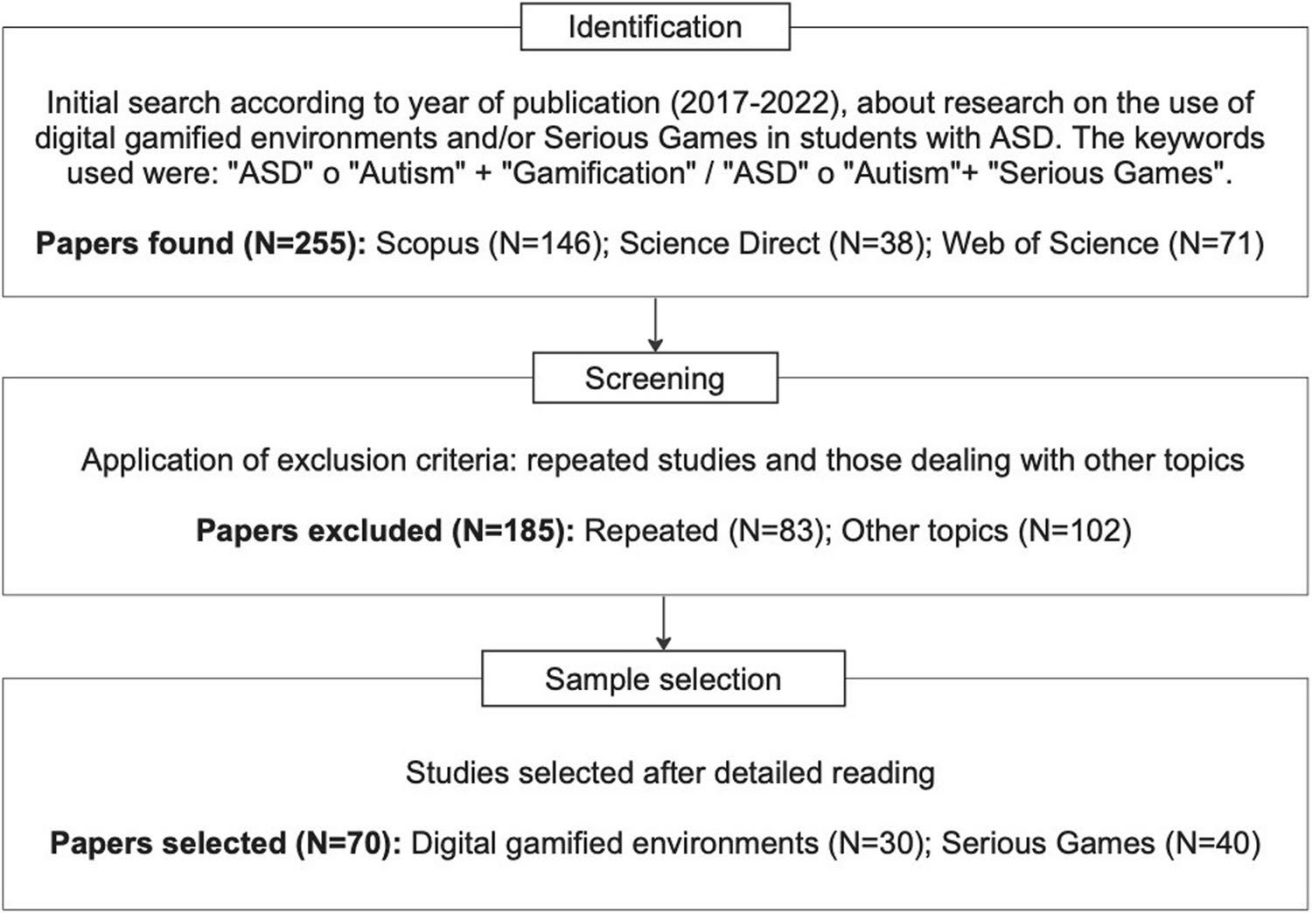
Research selection process. *Source*: own elaboration

All articles were examined, and an abstract review was carried out. Repeated investigations were excluded and those whose main content was other topics. Ultimately, the sample was made up of 70 studies. Sixteen systematic reviews were included, which although do not report specific experiences with subjects supported by digital gamified environments or Serious Games, provide the design keys for this type of resources based on expert opinions, prototype testing, etc. Figure 1 shows the research selection process.

**Fig. 2:**
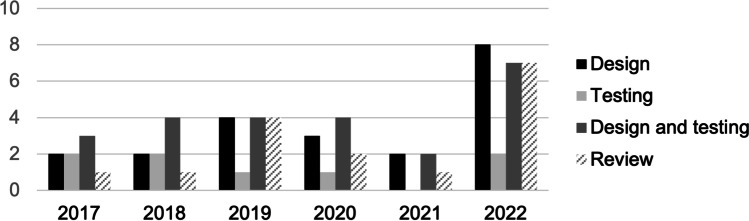
Sample distribution according to the publication period. *Source*: own elaboration

After carrying out the research quest following the pre-established selection criteria, a total of 70 studies were found: 30 on gamified environments and games and 40 on Serious Games. A thorough reading allowed for the extraction of authorship, nationality, publication period, and research design: method, main objectives, and procedure. Subsequently, adopting the SWOT analysis technique, following the criteria of Puyt et al. (2020), the strengths and weaknesses observed after the literature review were identified using a matrix that allowed for visualization and comparison. Its publication period is located, for the most part, in the period of 2022 (34.2%) (Fig. 2).

This map (Google Earth: http://bit.ly/3kcHeIP) shows the geographical organization of the universities and research groups which constitute the sample, locating most of the research on Gamified Environments in the American continent and, on Serious Games, in Europe.

**Fig. 3:**
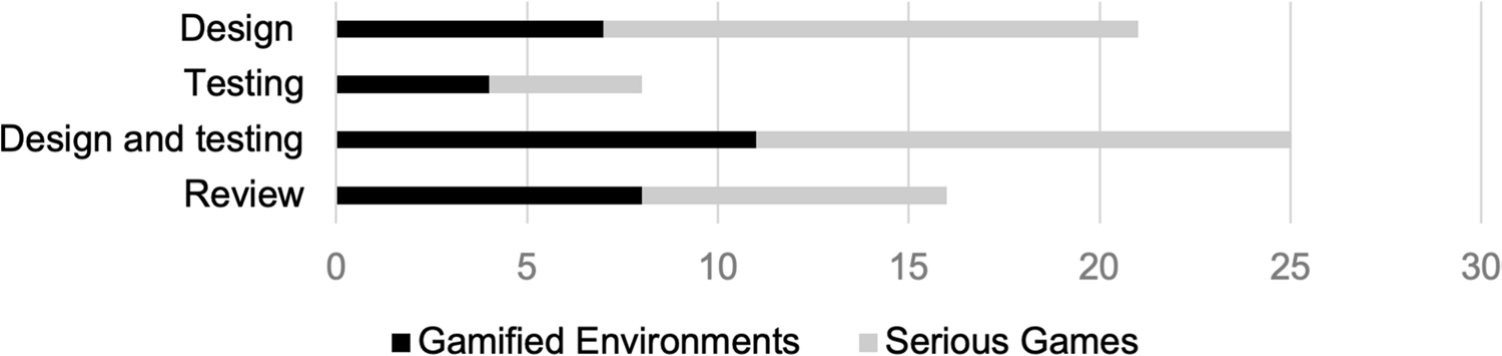
Distribution of the sample according to the kind of investigation. *Source*: own elaboration

Specifically, the studies are grouped around three study methods: the majority (35.7%) are framed in the design and testing of prototypes, followed by research focused just on their design (30%), theoretical reviews (22.8%) related to the search for elements, resources and strategies that favor greater stimulation of learning, and those studies focused on testing (11.4%), analyzing their reliability and validity (Fig. 3).

**Fig. 4:**
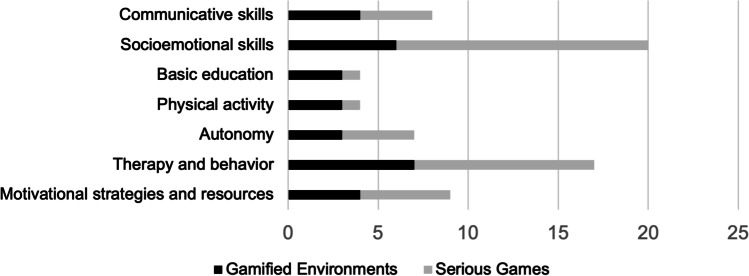
Distribution of the sample according to the object of study. *Source*: own elaboration

Trends are also observed within the object of study. Most of the research focuses on increasing socio-emotional skills (28.5%), followed by those that emphasize the use of Gamified Environments or Serious Games as therapeutic tools or focused on changes in behavior (24.2%) (Fig. 4).

## Results

### Gamified Environments and Students With ASD

Table 1 shows the studies on interventions with gamified digital environments in students with ASD.

**Table 1:** Studies on gamified digital environments and students with ASD

Authorship (date). Nationality		Design of the research	
	Method	Aims	Procedure
Adjorlu and Serafin (2019). Denmark.	Design	Develop skills in children and teenagers to cross the street properly.	Creation of a virtual environment co-designed by four teachers.
Arzone et al. (2020). Malaysia.	Review	Study the role of gamified environments to increase emotional intelligence (EI).	Literature review to establish guidelines for designing environments to improve EI.
Camargo et al. (2019). Brazil.	Review	Review the suitability of resources to develop gamified software.	Analysis of the resources, methods, and strategies used in 30 academic papers.
Constain et al. (2019). Colombia.	Review	Identify guidelines to design gamified environments which activate linguistic skills.	Review of techniques used and software design models.
Dantas and Nascimento (2022). Brazil.	Design and testing	Increase socio-emotional skills.	Design and testing of an environment that stimulates socio-emotional skills.
Elshahawy et al. (2022a). Egypt.	Design and testing	Stimulate problem solving.	Design, testing, and guidelines for interface design.
Goswami et al. (2021). India.	Design	Design a gamified environment to improve communicative skills.	Creation of activities which promote memory and vocabulary.
Hernández et al. (2020). Spain.	Design and testing	Improve the planning of children in medical consultations through gamification.	Creation of software that schedules medical appointments and allows communication in them.
Kalantarian et al. (2018). USA.	Design and testing	Create a gamified environment to diagnose disorders in behavior.	Design and testing of the game with 13 subjects with ASD. Viability analysis of the prototype.
Kim et al. (2020). USA.	Design and testing	Improve the physical activity of adults with ASD.	Design of a game and testing of the prototype (*N* = 229).
Lee et al. (2018). USA.	Design	Design a gamified app to increase physical activity.	Design of the prototype supported by theoretical review and needs analysis.
Lee et al. (2020). USA.	Testing	Evaluate the usability of a gamified app based on theory about changes in behavior.	Testing of the app with the intervention of 18 subjects through interviews.
Lee et al. (2022). USA.	Testing	Test the PuzzleWalk app and Google Fit.	Testing of the apps with 26 subjects for 5 weeks.
Li et al. (2018). USA.	Design and testing	Improve executive function (flexibility and cognitive function) through play.	Design and testing of a mobile game that uses social stimuli with 65 subjects.
Lu et al. (2022). China.	Design	Improve early intervention in the family of children with ASD.	Design of a gamified early care plan.
Luongo et al. (2022). Italy.	Design	Detect early motor problems.	Game-based software design that measures movement difficulties.
Malinverni et al. (2017). Spain.	Design and testing	Design encouraging and effective gamified environments in therapeutic terms.	Creation and testing of a game in 10 little children for their social initiation.
Mohammed and Aeisa (2017). Egypt.	Testing	Determine whether gamification replaces sensory integration training or not.	Testing of an app with 30 subjects in 30-min sessions for 6 months.
Mota et al. (2020). Brazil.	Design and testing	Implement a gamified environment with augmented reality.	Theoretical review, intervention, evaluation, and validation of the environment.
Mubin and Poh (2019). Malaysia.	Review	Improve social skills from gamified environments.	Identification and classification of the methods used.
Mubin et al. (2020). Malaysia.	Review	Review studies on the design of gamified environments.	Review of 6 clinical, immersive, and personalized studies.
Nabie and Gharebaghlou (2022). Iran.	Review	Review examples of gamification in urban environments.	Identification of suggestions to adapt the urban environment to people with ASD.
Najeeb et al. (2020). Sri Lanka.	Design and testing	Teach basic knowledge from a gamified game.	Design of the environment, intervention with monitoring, and evaluation.
Ntalindwa et al. (2022). Rwanda.	Review	Analyze the feasibility of incorporating gamified resources for learning mathematics.	Interviews with 56 teachers about the contents and methods used.
Pereira and Barwaldt (2022). Brazil.	Design	Stimulate geometric thinking.	Integration of gamified activities on a website.
Pires et al. (2022). Portugal.	Design and testing	Improve emotional recognition skills.	Creation and testing of a gamified software with a subject.
Silva et al. (2018). Brazil.	Testing	Motivate students to act and collaborate with their peers.	Game test that integrates collaborative tasks with 7 subjects.
Simões et al. (2022). Portugal.	Review	Review studies on the design of gamified experiences.	Literature review and identification of motivational keys.
Wang et al. (2022). China.	Design	Stimulate emotional understanding.	Software design to facilitate therapies
Wendt et al. (2020). USA	Design and testing	Create a digital game that stimulates language skills.	Review of research, testing, and usability tests.

*Source*: own elaboration

Most of the research is framed in the *design and testing of prototypes* (36.6%). Kalantarian et al. (2018) design and test the gamified game *Guess What?* in order to diagnose behavioral problems in people of this group. Malinverni et al. (2017) create *Pico’s Adventures* to promote social skills by implementing game mechanics and dynamics in a motivating environment. Along the same lines, Li et al. (2018) create a mobile game to stimulate cognitive function and social skills. Others opt for the creation of much more specific systems: Mota et al. (2020) implement *AssociAR*, a gamified project with augmented reality to associate images with words. Najeeb et al. (2020) present *Aliza*, a gamified smart mirror that includes literacy, verbal literacy, math, and emotional education components. Lastly, Wendt et al. (2020) enhance the autonomy of these people by creating the *SPEAK! Lite* and *SPEAKplay!* to stimulate speech in early ages and language, while training manipulation to access touch devices: touch, hold, drag, drop, and swipe.

Kim et al. (2020) design the *PuzzleWalk* app to stimulate physical activity in adults. Lee et al. (2022) test the feasibility of this app together with *Google Fit* to promote physical activity and eradicate a sedentary lifestyle. Pires et al. (2022) create an interface that seeks to stimulate facial recognition through neurofeedback. Dantas and Nascimento (2022) create a gamified environment that encourages the recognition and expression of basic emotions. Hernández et al. (2022) create the *PlanTEA* software, which allows planning medical appointments and communicating with specialists based on gamified activities. Finally, Elshahawy et al. (2022a) design and evaluate different gamified platforms to encourage problem solving using basic programming.

Regarding *reviews* (26.6%), Arzone et al. (2020) study the link between emotional intelligence and gamification as a source of learning for students with ASD. Constain et al. (2019) study the state of the art in relation to the use of gamification techniques in the development of applications. Camargo et al. (2019) and Mubin and Poh (2019) review the suitability of resources and methods included in the design of gamified environments for people from this group. Mubin et al. (2020) analyze the advantages of gamification to stimulate social skills in people with ASD. Nabie and Gharebaghlou (2022) analyze examples of gamification in urban environments adapted to people with ASD and specify suggestions to facilitate peer play. Simões et al. (2022) carry out a literature review to identify the key motivations of these environments. Furthermore, Ntalindwa et al. (2022) study the feasibility of integrating gamified resources in the Rwandan context.

Other studies focus on the *design of prototypes* (23.3%), observing a tendency to seek resources to stimulate interpersonal relationships, increasing linguistic-communicative skills or autonomy as a whole. Adjorlu and Serafin (2019) prioritize the development of skills to travel through the city. Wang et al. (2022) create software to facilitate emotional recognition during therapeutic sessions. Goswami et al. (2021) design *Dr. Memory*, where an eccentric scientist threatens to take over the world and only the player can stop him. This game stimulates memory by increasing the vocabulary necessary to go to a doctor’s office. Pereira and Barwaldt (2022) integrate activities on the code.org platform to facilitate the acquisition of knowledge about geometry. Lee et al. (2018) designed *PuzzleWalk*, an interactive system that incorporates gamified activities and augmented reality resources to promote physical exercise as a healthy habit in people from this group. Luongo et al. (2022) design a game-based software that allows them to be stimulated and detect early motor problems. On the other hand, Lu et al. (2022) improve the quality of early care by creating a gamified proposal for families.

Other studies aim attention at the description of the *testing of prototypes* (13.3%) created for these people. Lee et al. (2020) evaluate *PuzzleWalk*, a gamified mobile app to increase physical activity and reduce sedentary behavior in adults with ASD. Mohammed and Aeisa (2017) determine whether gamification could replace multisensory social integration training or not. Finally, Silva et al. (2018) analyze whether the *CoASD* environment favors collaborative tasks in the classroom.

### Serious Games and Students With ASD

As for Table 2, it presents research on interventions with students with ASD in gamified digital environments.

**Table 2:** Research about Serious Games to students with ASD

Authorship (date). Nationality		Design of the research	
	Method	Aims	Procedure
Almurashi et al. (2022). Saudi Arabia.	Review	Literature review on augmented reality, serious games, and PECS.	Search and comparison of 55 studies.
Antunes and Madeira (2022). Portugal.	Design	Improve the commitment and motivation of these people in therapeutic sessions.	Platform design that allows creating games applicable to specific therapies.
Barajas et al. (2017). Canada.	Design and testing	Create a tool which improves social and cognitive abilities.	Testing of a SG supported by lego blocks.
Bossavit and Parsons (2018). Spain.	Testing	Analyze the efficiency of an SG designed for teaching geography contents.	Pilot study which explores and analyzes a SG during 5 sessions.
Carlier et al. (2020). Belgium.	Design	Evaluate the viability of an SG in order to reduce stress and anxiety in students and their families.	Creation of a SG with minigames with relaxation techniques.
Carolis and Argentieri (2020). Italy.	Design	Improve physical activity and reduce these people’s risk of drowning.	Design of an SG to improve aquatic motor skills.
Carvalho et al. (2022). Brazil.	Design	Investigate how SGs have been evaluated before using them.	Review of OS evaluation criteria.
Chien et al. (2022). Taiwan.	Design	Stimulate gaze tracking, emotional recognition, and social interaction.	Design of a social interaction platform based on SG.
Dapogny et al. (2018). France.	Design	Stimulate recognition and production of facial expression in context.	Design of a SG that simulates social situations supported by virtual reality.
Derks et al. (2022). Holland.	Design and testing	Improve adaptive and cognitive functioning.	Design and testing of a SG to improve social participation with 654 subjects.
Elshahawy et al. (2020). Egypt.	Design	Stimulate problem solving derived from a SG prototype.	Design of a prototype to solve problems using computational thinking.
Elshahawy et al. (2022b). Egypt.	Design and testing	Help cope with changes during confinement.	Design of a prototype that promotes healthy habits.
Fridenson et al. (2017). Israel.	Testing	Testing a SG designed for emotional recognition.	Intervention from 6 to 9 years old for 8 to 12 weeks (*N* = 98).
Gaudí et al. (2019). Canada.	Design	Create an interface that will allow them to develop interventions with SG.	Design of two game examples.
Gómez et al. (2018). Norway and Spain.	Design and testing	Promote early literacy.	Pilot study with 9 subjects using a SG that promotes global reading.
Griffin et al. (2021). USA.	Design and testing	Stimulate gaze interpretation in the context of emotional recognition.	Intervention of 30-min sessions for 3 weeks with a SG (*N* = 34).
Grossard et al. (2017). France.	Review	Revision of literature about SG to teach social rules.	Search for studies (*N* = 31) and analysis of opportunities and limitations.
Grossard et al. (2019a, b). France.	Design	Design a SG which arouses emotional recognition.	SG design to recognize emotions in specific social situations.
Hassan et al. (2021). Germany.	Review	Analyze guidelines in the design of SG which improve behavior.	Revision of literature (*N* = 40) and creation of recommendations for future interventions.
Jaramillo et al. (2022). Ecuador.	Design	Design a method to evaluate the usability and accessibility of SG.	SG app design proposal.
Jiménez et al. (2022). Spain.	Review	Review the feasibility of using SG to reduce the degree of ASD.	Literature review and comparison between results with 24 studies.
Khowaja et al. (2018a). Malaysia.	Testing	Testing a SG prototype which helps in vocabulary learning.	Survey to teachers, prototype design, and intervention.
Khowaja et al. (2018b). Qatar.	Review	Revise the components for the design of SG to increase vocabulary.	Revision of literature and identification of components.
Mairena et al. (2019). Spain.	Design and testing	Compare the amount of social initiation behaviors during the game with SG.	Observational study (*N* = 15) of the interactions during the game.
Mercado et al. (2019). Mexico.	Design and testing	Improve attentional capacity in therapeutic sessions through neurofeedback.	Design and testing of the prototype that enhances sustained attention with 12 subjects.
Mora et al. (2017). Spain.	Design and testing	Encourage social interaction and collaborative behaviors.	SG design and control group testing.
Panceri et al. (2022). Brazil.	Design	Improve psychosocial therapies.	Development of a robot that integrates SG.
Papoutsi et al. (2022). Greece.	Review	Review SG to stimulate emotional intelligence.	Literature review and suggestion of new lines with 14 subjects.
Reyes et al. (2019). Ecuador.	Design and testing	Stimulate interpersonal relationships in children between 5 and 10.	Creation of a SG and testing with a case study.
Scherf et al. (2018). USA.	Design and testing	Increase understanding of gaze signals and attention to faces.	Design of a SG and intervention with teenagers (*N* = 34) for 3 weeks.
Silva et al. (2017). Brazil.	Design	Reduce the social isolation tendency.	Virtual SG design for the development of knowledge and social interaction.
Silva et al. (2019). Brazil.	Testing	Make the teaching-learning process easier and reduce the tendency of isolation.	Testing of SG with Multiagent System from questionnaires.
Taib et al. (2019). Singapore.	Design	Train executive skills.	SG design that facilitates planning routes in supermarkets.
Tang et al. (2019). Australia.	Design and testing	Identify the characteristics of motivation and learning for the creation of SG.	Intervention with a SG (*N* = 11) aimed at emotional recognition.
Terlouw et al. (2021). Holland.	Design and testing	Describe the process of designing a SG that facilitates communication.	Intervention in 4 sessions with two prototypes (*N* = 37).
Tsikinas and Xinogalos (2019). Greece.	Review	Determine the design principles for the creation of SG.	Review of literature and establishment of design guidelines and principles.
Vallefuoco et al. (2021). Italy.	Design	Train useful skills to recognize and discriminate coins and bills in real situations.	SG design with two 3D games set in real environments.
Vallefuoco et al. (2022). Italy.	Design and testing	Train the social skills necessary to make the purchase.	Design and testing of SG with virtual reality with 10 subjects.
Zakari et al. (2017). UK.	Design	Help moderate sensory hypersensitivity.	Creation and implementation of a SG which reduces hearing sensitivity.

*Source*: own elaboration

After the compilation of research, there is a heterogeneity between the different fields of study observed, although there is a greater predominance of research on design and focused on the design and testing of prototypes. In terms of *design* (35%), Gaudi et al. (2020) create the *ASGF* interface to develop interventions with SG in therapeutic sessions. In the same line, Jaramillo et al. (2022) establish the criteria to evaluate the usability and accessibility of the SG. The rest of the studies are structured around specific topics:

1. Communicative field: (a) linguistic, Silva et al. (2019) design *SEMATIC*, a SG created to stimulate reading and writing skills. And (b) socio-emotional, Dapogny et al. (2018) create *JEMImE* to stimulate socio-emotional skills from scenarios of gradual difficulty, which hybridize training and development of social skills in specific contexts. Subsequently, they improve the prototype, managing to encourage the production of facial expressions according to specific social situations (Grossard et al., 2019a, b). Panceri et al. (2022) develop a robot that integrates SG to improve psychosocial therapies. Chien et al. (2022) designed a game-based social interaction platform that stimulates gaze tracking, emotional recognition, and social skills.
2. Self-regulation skills: Carlier et al. (2020) create *New Horizon* to empower people in this group, reducing their stress and anxiety through mini-games which include gamification techniques. Elshahawy et al. (2020) present the *ADDIE* model, a SG that stimulates computational thinking in order to facilitate the everyday conflicts solving. Zakari et al. (2017) design *Sinbad* and *The Magic Cure* to moderate sensory hypersensitivity.
3. Skills for specific situations: Carolis and Argentieri (2020) design *iBall to Swim*, which presents activities in an aquatic environment to improve motor skills and learn basic swimming notions. Taib et al. (2019) evaluate a prototype to plan supermarket routes, stimulating this way executive function. Vallefuoco et al. (2021) design *€UReka* with the aim of training useful skills in adolescents to recognize and discriminate coins and notes to use them in real-life situations. Furthermore, Antunes and Madeira (2022) created a game design platform for therapeutic sessions, which allows sharing the results and comparing them in those clinics that use it.

Research focused on the *design and subsequent testing* of OS (35%) is also relevant, specifically linked to the stimulation of communication skills: on the one hand, associated with language skills, as pointed out by Gómez et al. (2018), by creating Leo con Lula, an app that favors reading literacy based on global reading methods. And, on the other hand, there are studies related to the increase in socio-emotional skills: Barajas et al. (2017) evaluate the usefulness of a SG built from physical building blocks — similar to Lego — augmented with electronic modules to enrich the therapy to improve social and cognitive skills. Mercado et al. (2019) create the *FarmerKeeper* prototype supported by the neurofeedback technique to stimulate sustained attention during the search for lost animals. Derks et al. (2022) create an SG to stimulate adaptive and cognitive-social behavior.

Griffin et al. (2021) create *Social Games for Autistic Adolescents (SAGA)* to increase sensitivity to gaze signals based on an immersive story that encourages interaction with animated characters and allows them to discover the usefulness of observing gaze signals to guide their behavior. In this same line, Scherf et al. (2018) create a SG that trains the people of this group to understand the changes in the gaze in order to facilitate their interpersonal relationships. Others focus on issues of health and autonomy: Elshahawy et al. (2022b) design a prototype to acquire behavior patterns during confinement derived from COVID-19, and Vallefuoco et al. (2022) design a SG to simulate the purchase through activities supported by virtual reality.

Mora et al. (2017) and Mairena et al. (2019) design Lands of Fog, a multiuser experience designed to encourage social initiation and collaborative behaviors. Reyes et al. (2019) develop the JOINME game to promote interpersonal relationships. Tang et al. (2019) carry out an investigation in order to identify the key characteristics for learning motivation during an intervention with a SG to recognize emotions. Terlouw et al. (2021) describe a SG based on Escape Room techniques whose objective is to facilitate direct communication between children with ASD and their peers.

Other papers are related to the *testing of prototypes* (10%) where the study areas are diverse. Bossavit and Parsons (2018) evaluate a resource to teach Geography and History to teenagers from this group. Fridenson et al. (2017) analyze *Emotiplay*, a system for emotional recognition with attractive aesthetics that favors student motivation. Khowaja et al. (2018a) test a prototype that activates vocabulary learning from simple images, while Silva et al. (2017) focus on the testing of *Knowledgemon Hunters*, a SG that aims to reduce the isolation tendencies of children with ASD through geolocation and virtual reality activities.

Finally, regarding studies focused on *theoretical reviews* (20%), Almurashi et al. (2022) and Jiménez et al. (2022) conduct a review of the use of SG in order to identify successful strategies for future interventions. More concretely, Grossard et al. (2017) focus on analyzing SGs that favor the social interaction of people with ASD, describing the design guidelines. Khowaja et al. (2018b) study the most suitable components of a SG to stimulate the acquisition of vocabulary. Hassan et al. (2021) focus on the design of SG to improve social behavior and Papoutsi et al. (2022) focus on the stimulation of emotional intelligence. Carvalho et al. (2022) analyze the evaluation criteria to select optimal SG for the interventions, how they have been applied, and what quality aspects have been evaluated. Tsikinas and Xinogalos (2019) establish the guidelines that should be taken into account in the creation of this type of tools aimed at interventions with people from this group using adults with ASD as testers.

## Discussion and Conclusions

Regarding the *gamified environments* in people with ASD, all the papers emphasize the great advantages of using the mechanics and dynamics of the game at the service of learning of people of this group, since an essential aspect of the success of the game is the attraction and motivation that lies beneath it (Malinverni et al., 2017). Specifically, it is observed that structured digital environments where the game is organized visually favor the assimilation and significance of the experience, generating greater learning (Lozano et al., 2012; Wang et al., 2022). Thus, the gamification of simple activities in digital environments raises expectations and provides rewards after their execution, which allows progress to be made at a slower pace (Kientz et al., 2013). In addition, these environments can promote self-awareness, self-control, motivation, empathy, and social skills (Arzone et al., 2020; Derks et al., 2022), in other studies also train interpersonal relationships and game skills (Nabie & Gharebaghlou, 2022), or emotional recognition (Pires et al., 2022), among others.

The rules of the game, the visual rewards, the feedback, and the establishment of short-term goals involve the students in the game from their intrinsic motivation (Lee et al., 2018). These environments can also favor autonomy (Camargo et al., 2019), problem solving (Elshahawy et al., 2022a), and monitoring of the evolution of students by families and teachers (Goswami et al., 2019; Lu et al., 2022) as well as physical activity (Lee et al., 2022; Luongo et al., 2022). In this context, it is important to integrate gamified techniques in clinical or psychoeducational treatments, especially when the aim is to develop skills such as self-recognition and social performance (Constain et al., 2019). Ntalindwa et al. (2022) underline the opportunity that these environments present when appealing to centers of interest for children. In addition, the incorporation of augmented reality in these gamified environments can favor the interpretation of the incorporated simulations (Mota et al., 2020). Furthermore, Vallefuoco et al. (2022) use virtual reality to improve daily autonomy.

Regarding the limitations observed in the gamified environments used in the interventions with these subjects, Lee et al. (2020) point out that they do not usually adapt to the social and health needs of adulthood (world of work, sentimental relationships, etc.) Malinverni et al. (2017) point out that there are no mental health experts involved in the design of these environments, nor are the interests of minors or the experience of the designers considered, so it would be interesting to create interdisciplinary teams. On the other hand, Camargo et al. (2019) highlight that intervention with these environments is a challenge, since the complex clinical conditions and the wide range of symptoms that the disorder covers complicate the interaction with these resources. Thus, the extrapolation of interventions to multiple contexts is complex due to the heterogeneity of characteristics of people with ASD (Mota et al., 2020). In addition, it is essential to start from previous research related to the keys to design and take into account the results of previous tests to optimize the design of gamified resources to achieve pre-established aims (Mubin & Poh, 2019). Therefore, the usability plan of these environments (Jaramillo et al., 2022) and the prior selection guidelines (Carvalho et al., 2022) are decisive in favoring the usability of these environments (Lee et al., 2018).

On the other hand, with reference to the use of *Serious Games* in interventions with people with ASD, it is observed that these can be heterogeneous and used in various ways, favoring social interactions in several contexts and situations such as during the pandemic (Elshahawy et al., 2022b). Fridenson et al. (2017) suggest that scenarios can simulate real-world situations, reducing the cognitive load of identifying and internalizing social norms, and stimulating linguistic competence (Khowaja et al., 2018a, b). Furthermore, Tang et al. (2017) highlight the opportunity for GS for emotional recognition in a safe and autonomous environment, becoming a solution to reduce their feelings of frustration and anxiety. Specifically, Tang et al. (2019) highlight the importance of gradually increasing the difficulty of the tasks in the game and adjusting the demands of the game to the skills of the player.

Concerning the creation of SG, Terlouw et al. (2021) indicate that an interactive design encourages goal achievement by implementing motivating and personalized chained activities that adapt to the rhythms of each player (Elshahawy et al., 2020). In addition, the use of SG in work groups reduces students’ solo play (Barajas et al., 2017). The audiovisual environments of the SG favor learning, retention, and fixation of information in people with ASD, since they tend to retain visual information better (Elshahawy et al., 2020; Khowaja et al., 2018a, b). In addition, the player can individualize his gaming experience, customizing some options according to his preferences and needs (Vallefuoco et al., 2021). This individualized approach allows it to be extrapolated both to therapeutic interventions and to use in the family or school context (Carlier et al., 2020; Mercado et al., 2019). For its part, the research by Tsikinas and Xinogalos (2019) has subjects with ASD to test a prototype, highlighting the need to integrate a variety of resources and scenarios that recreate real-life situations within a motivating narrative.

Regarding the limitations of the use of SG, Tang et al. (2019) highlight the evidentiary gender bias of the sample, since the game would be required to contemplate the points of view of women with ASD. Atherton and Cross (2021) point out that there are few studies supported by large samples and most have not shown how skills improved. Hassan et al. (2021) and Silva et al. (2018) conclude that, from a design perspective, SG testing should be carried out with a larger sample, as well as clinical validation and periodic follow-up. In addition, Hulusic and Pistoljevic (2017) indicate that there are few appropriate SGs for people with ASD in languages other than English. Jiménez et al. (2022) point out the need to design commercial SGs adapted to people in this group.

As future lines of research, and in accordance with Grossard et al. (2017), it is noted that most gamified and SG environments are developed for high-functioning individuals (Fridenson et al., 2017; Terlouw et al., 2021), requiring a line of research aimed at people with moderate ASD. Grossard et al. (2017) also point out that, occasionally, the clinical validation of gamified or SG environments and the testing of their playability are not compatible. Research is needed to conclude whether the potential of these resources is maintained over time or is only subscribed at the time of intervention (Grossard et al., 2019a, b; Terlouw et al., 2021). Finally, Grossard et al. (2019a, b) conclude that although there are several games related to the recognition of facial expressions, few focus on the production of facial expressions adapted to a given context.
